# Social Media as a Platform for Cancer Care Decision-Making Among Women: Internet Survey-Based Study on Trust, Engagement, and Preferences

**DOI:** 10.2196/64724

**Published:** 2025-03-05

**Authors:** Anna Rose Johnson, Grace Anne Longfellow, Clara N Lee, Benjamin Ormseth, Gary B Skolnick, Mary C Politi, Yonaira M Rivera, Terence Myckatyn

**Affiliations:** 1 Division of Plastic and Reconstructive Surgery Department of Surgery Washington University School of Medicine Saint Louis, MO United States; 2 Department of Surgery Lineberger Comprehensive Cancer Center University of North Carolina at Chapel Hill Chapel Hill, NC United States; 3 Department of Plastic Surgery University of Texas Southwestern Medical Center Dallas, TX United States; 4 Division of Public Health Sciences Department of Surgery Washington University School of Medicine Saint Louis, MO United States; 5 Department of Communication School of Communication & Information Rutgers University New Brunswick, NJ United States

**Keywords:** shared decision-making, SDM, decision aids, cancer treatment, breast cancer, digital health, social media, health communication, online decision aids, health information-seeking behavior, trust in health information, healthcare accessibility, mhealth

## Abstract

**Background:**

Decision aids improve patient and clinician decision-making but are underused and often restricted to clinical settings.

**Objective:**

Given limited studies analyzing the feasibility of disseminating decision aids through social media, this study aimed to evaluate the acceptability, trust, and engagement of women with social media as a tool to deliver online decision aids for cancer treatment.

**Methods:**

To prepare for potential dissemination of a breast cancer decision aid via social media, a cross-sectional survey in February 2023 was conducted via Prime Panels, an online market research platform, of women aged 35-75 years in the United States. Demographics, health, cancer information-seeking behaviors, social media use, trust in social media for health information, as well as the likelihood of viewing cancer-related health information and clicking on decision aids through social media, were assessed. Statistical analyses included descriptive statistics, correlations, and multivariable ordinal regression.

**Results:**

Of 607 respondents, 397 (65.4%) had searched for cancer information, with 185 (46.6%) using the internet as their primary source. Facebook (Meta) was the most popular platform (511/607, 84.2%). Trust in social media for health information was higher among Black (14/72, 19.4%) and Asian respondents (7/27, 25.9%) than among White respondents (49/480, 10.2%; *P*=.003). Younger respondents aged 35-39 years (17/82, 20.7%) showed higher trust than those aged 70-79 years (12/70, 17.1%; *P*<.001). Trust in social media for health information was linked to a higher likelihood of viewing cancer information and accessing a decision aid online (*P*<.001). Participants who rated social media as “Trustworthy” (n=73) were more likely to view cancer information (61/73, 83.6%) and click on decision aids (61/73, 83.6%) than those who found it “Untrustworthy” (n=277; view: 133/277, 48.0%; click: 125/277, 45.1%). Engagement with social media positively correlated with viewing online cancer information (Spearman ρ=0.20, *P*<.001) and willingness to use decision aids (ρ=0.21, *P<.001*). Multivariable ordinal regression analyses confirmed that perception of social media’s trustworthiness is a significant predictor of engagement with decision aids (untrustworthy vs trustworthy β=–1.826, *P*<.001; neutral vs trustworthy β=–0.926, *P*=.007) and of viewing cancer information (untrustworthy vs trustworthy β=–1.680, *P*<.001, neutral vs trustworthy β=–0.581, *P=.*098), while age and employment status were not significant predictors.

**Conclusions:**

This exploratory study suggests that social media platforms may increase access to health information and decision aids. No significant differences were observed between demographic variables and the use or trust in social media for health information. However, trust in social media emerged as a mediating factor between demographics and engagement with cancer information online. Before disseminating decision aids on social media, groups should identify existing trust and engagement patterns with different platforms within their target demographic.

## Introduction

An estimated 2,001,140 new cancer cases are expected in 2024 [1]. Shared decision-making (SDM) describes a process between the clinician and patient to facilitate preference-sensitive choices [2]. Decision aids, which can support the SDM process, are evidence-based tools designed to provide patients with information, clarify their preferences, and prepare them to make a choice [3,4]. In this study, we explore the potential of social media as an avenue for engagement with decision-making tools.

SDM has been shown to be important for cancer decision-making, with multiple randomized controlled trials demonstrating that decision aids improve patient knowledge and the quality of decisions [3-11]. Unfortunately, decision aid use has been limited, and their dissemination has been largely confined to clinical settings. A 2010 study revealed that only 24% of clinicians working with patients with cancer used decision aids [12-18]. The focus on clinical settings as the singular forum for decision aid deployment is predicated on clinician buy-in and may restrict the use of decision aids to a select cohort of the population [12-19].

Social media offers a promising means for disseminating decision aids without relying on health care access. It may also provide a more extended and personalized modality for disseminating information [20]. With 81% of Americans using social media, a number that continues to grow, social media platforms present an underused opportunity to disseminate highly accessible decision-making tools [21]. Social media can help to overcome challenges associated with traditional clinical encounters (ie, time, workflow, etc) and can enhance the patient-clinician relationship by promoting empowerment, reducing communication barriers, and increasing knowledge about conditions and treatment options [14,22].

Numerous studies have highlighted the positive impact of web-based decision aids for women, particularly in the context of breast cancer, the leading cause of cancer among females [23]. Despite the potential benefits of social media for decision aid dissemination, it remains uncertain whether females will use cancer-related decision aids available through social media or other online channels. To address this gap, we examined factors influencing engagement with decision aids on social media and explored health information-seeking behaviors across various platforms. This study aimed to evaluate the feasibility, acceptability, trust, and engagement with social media as a tool to deliver online decision aids to women for cancer treatment. By focusing specifically on women, we aimed to address the unique health and decision-making needs of this population and provide insight for future research on breast cancer-related decision aids.

## Methods

### Survey Design

A cross-sectional survey was designed to assess the use and preferences for social media–advertised decision-making tools for cancer care. The survey involved several key areas of inquiry (Multimedia Appendix 1). Briefly, these areas included “Health-Related Information Behaviors” (5 questions) to assess participants’ behaviors in seeking and using health information; “Sources of Health Information” (3 questions), exploring individuals’ preferences and trust levels in various health information sources; “Social Media Use” (18 questions), examining patterns and motivations behind social media interactions, particularly concerning health information; and “Demographic Data” (10 questions), covering a wide range of personal and socioeconomic factors. Within the “Social Media Use” section, two items related to the main outcomes of the study were embedded, created by the study team, which asked participants to imagine themselves or a loved one deciding about cancer treatment and then assessing their likelihood of viewing cancer treatment information or clicking on a decision aid posted on social media. Other survey questions were adapted from items from the Health Information National Trends Survey [24]. Items assessing reasons to use social media included categories identified in the literature through the uses and gratifications theory and the social media engagement model [25-27].

The response formats varied according to the specific inquiry, including multiple-choice options, checkboxes for applicable answers, and Likert scales for assessing attitudes and opinions. Although all 39 questions could be answered, branching logic was used to tailor the survey based on participants’ responses (eg, only those reporting the use of specific social media platforms were asked follow-up questions about their motivations for use). The survey was designed to be completed within 5 to 10 minutes and included 2 attention-check questions. One single question, with the associated branching logic, was shown on the screen at a time. Participants were unable to skip questions (except for the questions asking the frequency of use and reason for use of social media platforms) and were notified, “Please answer this question” if attempted to skip. Participants were able to go back and change their answers if desired. The order of survey items, and answers, was fixed and not randomized, as the survey design prioritized logical flow and ease of navigation for participants. This study is reported in a manner that is consistent with the specified Checklist for Reporting Results of Internet E-Surveys (CHERRIES) guidelines (Multimedia Appendix 2) [28].

### Population Targeting and Survey Distribution

The survey was designed and hosted on the Qualtrics platform (Qualtrics, Provo) and distributed in February 2023 via Prime Panels, a component of Cloud Research [29]. Prime Panels uses a novel data collection method by aggregation of diverse opt-in market research panels into a comprehensive sampling platform, facilitating the recruitment of participants from existing commercial panel pools. This method supports demographic quotas and specific eligibility criteria, enhancing data representativeness, especially among hard-to-reach populations. Eligible participants were invited to participate through targeted email and dashboard invitations sent by the market research panels within the Prime Panels network, based on the demographic criteria specified for the study [30]. Participants were required to complete the survey in a single sitting, and no reminders were sent to those who did not finish it during that session. Due to the wide distribution on several platforms, response rates and the total number of invitations sent were not calculated by Prime Panels.

We aimed to gather a representative sample of United States females aged 35-75 years for this study, as this age range represents the peak period for breast cancer diagnosis. The study exclusively enrolled female participants to direct focus toward future research efforts related to breast cancer in women. Using 2022 US Census data, the population of females aged 35-75 years were inputted into the Qualtrics sample size calculator with a 99% CI and a 5% margin of error to determine the required sample size [31]. Based on these calculations, the survey targeted approximately 660 female participants, with an additional 15% included to account for potential exclusions due to poor response quality, bringing the total recruitment goal to 750 participants. Study specific eligibility criteria incorporated into the Prime Panels query included female participants aged 35 years or older with US IP addresses. Demographic quotas based on United States Census Bureau parameters were set as: 16% Hispanic, Latino, or Spanish origin; 78% White; 13% Black; 5% Asian; 2% American Indian or Alaskan Native; and 2% other races. Age quotas aimed for an equal distribution between the 35-55 years and 56-75 years age ranges to reflect the demographics of breast cancer survivors. To ensure survey security, Qualtrics options for “Bot Detection,” “RelevantID,” and “Prevent Indexing” were enabled.

### Statistical Analyses

Data analysis was performed using Microsoft Excel and SPSS Statistics (version 28.0; IBM Corp). Graphs were constructed via R Statistical Software (version 4.1.2; R Core Team 2021). Descriptive statistics characterized the demographic characteristics, health-seeking behaviors, and social media engagement of the study population. Social media engagement was determined based on respondents’ selections of platforms they actively used, followed by survey questions that assessed the frequency of engagement with each platform. These questions categorized usage frequency into 4 levels: multiple times a day, once a day, at least 3 times a week, or less than 3 times a week. Numerical values ranging from 1 to 4 were assigned to these categories, with 1 indicating the least frequent usage and 4 the most frequent. An “Overall Social Media Engagement Score” was computed by aggregating these values across all platforms used by a respondent. Participants were classified into 4 groups based on their “Overall Social Media Engagement Score” to approximate quartiles for analysis. These groups were defined as follows: “Low Engagement” (scores of >0 and ≤3), “Moderate Engagement” (scores of >3 and ≤4), “Moderate-High Engagement” (scores of >4 and ≤8), and “High Engagement” (scores of >8 and ≤23).

To ensure data integrity, surveys were excluded based on the following criteria: completion times shorter than 3 minutes or longer than 20 minutes, flags from Qualtrics indicating duplicate responses, an Amazon Mechanical Turk fraud score above 50, or incorrect responses to two embedded control multiple-choice questions.

#### Analysis of Trustworthiness of Social Media

To evaluate associations between demographic factors and the perceived trustworthiness of social media as a reliable source of health information, chi-square, and Fisher’s exact tests were performed. To ensure that there were enough observations in each category for the statistical analysis to be reliable, the response categories “Trustworthy” and “Very Trustworthy” were merged into a single “Trustworthy” category, while “Untrustworthy” and “Very Untrustworthy” were combined into “Untrustworthy.”

#### Analysis of Social Media Engagement

In terms of the two questions assessing the likelihood of engaging with cancer treatment-related information seen on social media, responses were condensed from a 7-tier scale to 3 categories: “Unlikely” (1-3), “Neutral” (4), and “Likely” (5-7) to ensure a more balanced distribution of responses, as some of the original categories had very few observations. Spearman rank correlation coefficients were calculated to quantify the strength and direction of the association between the “Overall Social Media Engagement Score” and the tiered scores representing the likelihood of interacting with cancer-related information. For visual interpretation, the mean likelihood of respondents interacting with cancer-related information was calculated for each unique “Overall Social Media Engagement Score.” For all analyses, statistical significance was set at a *P* value of less than .05, using 2-tailed testing.

#### Analysis of Likelihood to View Cancer-Related Health Information or Click on Decision Aid

Nonparametric tests, specifically the Kruskal-Wallis and Mann-Whitney *U* tests, were used to evaluate the relationships between demographic characteristics, trust in social media, and the propensity to use decision aids or view cancer-related health information on these platforms. Variables that were found to be significantly related to the use of decision aids or viewing health information at *P*≤.10 were then checked for multicollinearity via variance inflation factor (VIF) values <5 before inclusion in an ordinal regression model.

### Ethical Considerations

This study was reviewed and received approval from the institutional review board at Ohio State University as exempt (protocol 2022E0836). Informed consent was obtained from all participants involved in the study. Participant data were collected anonymously, with no identifying information retained in the dataset. The original informed consent included provisions for the use of deidentified data for research purposes, as reviewed and approved by the institutional review board. Data were stored in a secure, password-protected database accessible only to study investigators. All participants were compensated by Prime Panels in the amount agreed to by the platform through which they entered the survey, which is unknown to study personnel.

## Results

A total of 757 responses were initially recorded at the completion of distribution. Of these, 607 met inclusion criteria with a Qualtrics “Response Quality” of 99.0%. Participants completed the survey in a mean SD time of 5.5 (SD 2.6) minutes.

### Respondent Demographics

All participants were female, aged 35-75 years (Table 1). Of the 607 respondents, most were non-Hispanic (556/607, 91.6%) and White (480/607, 79.1%). The most common education level was some college or an associate degree (201/607, 33.1%). The most common income range was US $20,000 to US $35,000 (119/607, 18.9%), with over half (327/607, 53.9%) earning less than US $50,000 annually.

**Table 1:** Demographic characteristics of respondents.

Characteristics		Respondents (N=607), n (%)
**Ethnicity**		
	Hispanic	48 (7.9)
	Non-Hispanic	556 (91.6)
	Unknown or prefer not to answer	3 (0.5)
**Race**		
	White	480 (79.1)
	Black	72 (11.9)
	Asian	27 (4.4)
	Native American or Alaskan Native	16 (2.6)
	Native Hawaiian or Pacific Islander	0 (0.0)
	Other or prefer not to answer	11 (1.8)
**Age (years)**		
	35-39	82 (13.5)
	40-49	141 (23.2)
	50-59	149 (24.5)
	60-69	165 (27.2)
	70-79	70 (11.5)
**Highest level of education achieved**		
	High school diploma, GED^a^, or less	175 (28.8)
	Technical training or certificate	46 (7.6)
	Some college or associate degree	201 (33.1)
	College degree	111 (18.3)
	Graduate or professional degree	74 (12.2)
**Annual household income (US $)**		
	<20,000	109 (18.0)
	20,000-35,000	115 (18.9)
	35,000-50,000	103 (17.0)
	50,000-75,000	109 (18.0)
	75,000-100,000	73 (12.0)
	>100,000	82 (13.5)
	Unknown or prefer not to answer	16 (2.6)
**Insurance type**		
	Private	229 (37.7)
	Government	301 (49.6)
	Uninsured	43 (7.1)
	Other	8 (1.3)
	More than 1 insurance policy	26 (4.3)
**Relationship status**		
	Single	129 (21.3)
	Married	268 (44.2)
	Separated or divorced	150 (24.7)
	Widowed	57 (9.4)
	Other or unknown or prefer not to answer	3 (0.5)
**Employment status**		
	Full-time	207 (34.1)
	Part-time	71 (11.7)
	Not working for pay or unemployed	121 (19.9)
	Retired	200 (32.9)
	Other or unknown or prefer not to say	8 (1.3)
**Country of birth**		
	United States	555 (91.4)
	Outside of the United States	52 (8.6)
**Years of US residency**		
	Less than 15 years	20 (3.3)
	More than 15 years	587 (96.7)

^a^GED: graduate educational diploma.

### Health-Seeking Behavior

In total, 551 out of 607 participants (90.8%) had sought health or medical information from various sources at some point (Table 2). Out of 551 respondents, the internet was the most common first source of health information (n=441/551, 80.0%), while 75 or 13.6% consulted a doctor or health care provider.

**Table 2:** Characteristics of health-seeking behaviors.

Question	Respondents (N=607), n (%)
Have you ever looked for information about health or medical topics from any source?	551 (90.8)
The most recent time you looked for information about health or medical topics, where did you go first?	551 (100)
Doctor or health care provider	75 (13.6)
Internet	441 (80)
Brochure or pamphlet, etc.	10 (1.8)
Friend or coworker	3 (0.5)
Family	11 (2)
Cancer organization	2 (0.4)
Newspapers	1 (0.2)
Books	5 (0.9)
Library	2 (0.4)
Telephone information number	1 (0.2)
The most recent time you looked for information about health or medical topics, who was it for?	551 (100)
Self	408 (74)
Someone else	72 (13.1)
Both oneself and someone else	71 (12.9)
Which of the following sources have you used in the last month as a source of news or information about health topics?^a^	592 (100)
Blogs or personal websites	72 (12.2)
Center for disease control and prevention	133 (22.5)
World Health Organization	63 (10.6)
Government	46 (7.8)
Community or faith leaders	19 (3.2)
Online news	256 (43.2)
Email	48 (8.1)
Family and friends	204 (34.5)
Health professionals	282 (47.6)
Radio	22 (3.7)
Podcasts	27 (4.6)
TV	69 (11.7)
Social media	119 (20.1)
Print media	46 (7.8)
Video sharing sites	53 (9)
Have you ever looked for information about cancer from any source?	607 (100)
Yes	397 (65.4)
No	210 (34.6)
In the past 12 months, have you used the internet to look for cancer information for yourself?	397 (100)
Yes	185 (46.6)
No	212 (53.4)
Where do you access your social media accounts?^a^	603 (100)
Computer or laptop	258 (42.8)
iPad or tablet	159 (26.4)
Smartphone	497 (82.4)

^a^Participants were able to check all that apply.

### Social Media Use and Engagement

In total, 80 out of 607 or 95.6% of respondents used social media. Of these, Facebook was the most popular platform, used by 511 or 84.2%, and was used primarily for social interactions by 338 out of 487 respondents (69.4%). YouTube (Alphabet Inc) and Instagram (Meta) were primarily used for entertainment (189 out of 251 or 75.3% and 110 out of 193 or 57.0%, respectively). 18.5%, or 112 out of 607 respondents, demonstrated a “Low Engagement” pattern regarding social media use.

### Trustworthiness of Social Media

The majority of the 607 respondents found social media trustworthy (73/607, 12.0%) or neutral (257/607, 42.3%) for health information. Black or Asian race, younger age, and longer duration of US residency were associated with greater trust in social media. Among Black respondents, 14 out of 72 (19.4%) considered social media trustworthy, compared to 49 out of 480 (10.2%) of White respondents (*P*=.003). Asian respondents showed even higher trust levels, with 7 out of 27 (25.9%) rating social media as trustworthy. Younger individuals also reported greater trust, with 17 out of 82 (20.7%) of those aged 35-39 years trusting social media compared with 12 out of 70 (17.1%) among those aged 70-79 years (*P*<.001). In addition, respondents with longer US residency (more than 15 years) showed greater trust, with 272 out of 587 (46.3%) indicating trustworthiness in social media, compared with only 5 out of 20 (25.0%) of those with less than 15 years of residency (*P=.*004). In total, 277 respondents (45.6%) noted social media to be untrustworthy (Table 3).

**Table 3:** Factors associated with perceived trustworthiness of social media as a source for health information.

Factors			Trustworthy		Neutral		Untrustworthy			Respondents, n (%)		*P* value	
Total			73 (12)		257 (42.3)		277 (45.63)		607 (100)			N/A^a^	
**Ethnicity**													.14
	Non-Hispanic	64 (11.5)		233 (41.9)		259 (46.58)		556 (100)					
	Hispanic	9 (18.8)		23 (47.9)		16 (33.33)		48 (100)					
	Unknown or prefer not to answer	0 (0.08)		1 (33.3)		2 (66.67)		3 (100)					
**Race**													.003^b^
	White	49 (10.2)		194 (40.4)		237 (49.4)		480 (100)					
	Black	14 (19.4)		32 (44.4)		26 (36.1)		72 (100)					
	Asian	7 (25.9)		15 (55.6)		5 (18.5)		27 (100)					
	Native American or Alaskan Native	2 (12.5)		7 (43.8)		7 (43.8)		16 (100)					
	Native Hawaiian or other Pacific Islander	0 (0)		0 (0)		0 (0)		0 (100)					
	Other or prefer not to answer	1 (8.3)		9 (75)		2 (16.7)		12 (100)					
**Age (years)**													<.001^b^
	35-39	17 (20.7)		33 (40.2)		32 (39)		82 (100)					
	40-49	20 (14.2)		68 (48.2)		53 (37.6)		141 (100)					
	50-59	13 (8.7)		71 (47.7)		65 (43.6)		149 (100)					
	60-69	11 (6.7)		67 (40.6)		87 (52.7)		165 (100)					
	70-79	12 (17.1)		18 (25.7)		40 (57.1)		70 (100)					
**Highest level of formal education achieved**													.40
	High school diploma, GED^c^, or less	24 (13.7)		81 (46.3)		70 (40)		175 (100)					
	Technical training or certificate	5 (10.9)		19 (41.3)		22 (47.8)		46 (100)					
	Some years of college or associate degree	23 (11.4)		92 (45.8)		86 (42.8)		201 (100)					
	College degree	12 (10.8)		40 (36)		59 (53.2)		111 (100)					
	Graduate or professional degree	9 (12.2)		25 (33.7)		40 (54.1)		74 (100)					
**Annual household income (US $)**													.63
	<20,000	11 (10.1)		53 (48.6)		45 (41.3)		109 (100)					
	20,000-35,000	17 (14.8)		48 (41.7)		50 (43.5)		115 (100)					
	35,000-50,000	15 (14.6)		47 (45.6)		41 (39.8)		103 (100)					
	50,000-75,000	12 (11)		43 (39.5)		54 (49.5)		109 (100)					
	75,000-100,000	10 (13.7)		24 (32.9)		39 (53.4)		73 (100)					
	>100,000	8 (9.8)		35 (42.7)		39 (47.6)		82 (100)					
	Unknown or prefer not to answer	0 (0)		7 (43.8)		9 (56.3)		16 (100)					
**Insurance type**													.16
	Private	28 (12.2)		98 (42.8)		103 (45)		229 (100)					
	Government	42 (14)		123 (40.9)		136 (45.2)		301 (100)					
	Uninsured	1 (2.3)		24 (55.8)		18 (41.9)		43 (100)					
	Other insurance or more than 1 policy	2 (5.9)		12 (35.3)		20 (58.8)		34 (100)					
**Relationship status**													.79
	Single	15 (11.6)		58 (45)		56 (43.4)		129 (100)					
	Married	33 (12.3)		102 (38.1)		133 (49.6)		268 (100)					
	Separated or divorced	18 (12)		70 (46.6)		62 (41.3)		150 (100)					
	Widowed	7 (12.3)		25 (43.9)		25 (43.9)		57 (100)					
	Other or unknown or prefer not to answer	0 (0)		2 (66.7)		1 (33.3)		3 (100)					
**Employment status**													.09
	Full-time	22 (10.6)		96 (46.4)		89 (43)		207 (100)					
	Part-time	10 (14.1)		31 (43.7)		30 (42.3)		71 (100)					
	Not working for pay or unemployed	17 (14.1)		58 (47.9)		46 (38)		121 (100)					
	Retired	24 (12)		68 (34.0)		108 (54)		200 (100)					
	Other or unknown or prefer not to say	0 (0)		1 (50)		1 (50)		2 (100)					
**Country of birth**													.28
	United States	64 (11.5)		233 (41.98)		258 (46.49)		555 (100)					
	Outside of the United States	9 (17.3)		24 (46.15)		19 (36.54)		52 (100)					
**Years of US residency**													.004^b^
	Less than 15 years	7 (35)		8 (40)		5 (25)		20 (100)					
	More than 15 years	66 (11.2)		249 (42.4)		272 (46.3)		587 (100)					
**Platforms used (multiple selections allowed)**													N/A
	Facebook	65 (12.7)		220 (43.1)		226 (44.2)		511 (100)					
	Twitter (rebranded as X)	23 (16.9)		65 (47.8)		48 (35.3)		136 (100)					
	Instagram	35 (12.6)		125 (45.1)		117 (42.2)		277 (100)					
	YouTube	53 (13)		179 (45.4)		162 (41.1)		394 (100)					
	WhatsApp (Meta)	17 (21.3)		34 (42.5)		29 (36.3)		80 (100)					
	TikTok (ByteDance)	22 (12.9)		85 (49.7)		64 (37.4)		171 (100)					
	Other or unknown	4 (13.3)		10 (33.3)		16 (53.3)		30 (100)					

^a^N/A: not applicable.

^b^*P*<.05.

^c^GED: graduate educational diploma.

Among social media platforms, the highest proportion of trustworthy users was noted among the 80 WhatsApp users (n=17, 21.3%), followed by 23 out of the 136 (16.9%) Twitter users. Amongst the 511 respondents who used Facebook, the most frequently used platform, 65 or 12.7% reported trust in social media for health information.

### Use of Cancer Information or Decision Aids Through Social Media

Participants who considered social media “Trustworthy” (n=73) were more likely to view cancer information (n=61, 83.6%) or click on a decision aid through social media (n=61, 83.6%) than the 277 respondents who viewed social media as “Untrustworthy” (view: n=133, 48.0%; click: n=125, 45.1%) (Tables 4 and 5). Younger participants, particularly those aged 35-39 years were more likely to view cancer-related information through social media. Only 10 out of 57 (12.2%) in the 35-39 years age group rated their likelihood as “unlikely,” compared with 54 out of 89 (32.7%) aged 60-69 years. Among respondents aged 35-39 years, 54 out of 82 (65.9%) were likely to click on the decision aid, while in the 60-69 years age group, 87 out of 165 (52.7%) indicated they were likely to click.

**Table 4:** Nonparametric analysis of factors influencing viewing of cancer-related health information on social media: social media trustworthiness and demographic insights.

Variable		Likelihood of viewing cancer-related health information seen on social media					Respondents, n (%)			*P* value	
		Unlikely	Neutral	Likely							
Total		141 (23.23)	82 (13.51)	384 (63.26)		607 (100)			N/A^a^		
**Trustworthiness of social media**											<.001^b^
	Untrustworthy	99 (35.7)	45 (16.2)	133 (48)		277 (100)					
	Neutral	36 (14)	31 (12.1)	190 (73.9)		257 (100)					
	Trustworthy	6 (8.2)	6 (8.2)	61 (83.6)		73 (100)					
**Ethnicity**											.86
	Hispanic	130 (23.4)	75 (13.5)	351 (63.1)	556 (100)						
	Non-Hispanic	11 (22.9)	6 (12.5)	31 (64.6)	48 (100)						
	Unknown or prefer not to answer	0 (0)	1 (33.3)	2 (66.7)	3 (100)						
**Race**											.17
	White	110 (22.9)	68 (14.2)	302 (62.9)		480 (100)					
	Black	23 (31.9)	9 (12.5)	40 (55.6)		72 (100)					
	Asian	4 (14.8)	2 (7.4)	21 (77.8)		27 (100)					
	Native American or Alaskan Native	2 (12.5)	3 (18.8)	11 (68.8)		16 (100)					
	Native Hawaiian or Pacific Islander	0 (0)	0 (0)	0 (0)		0 (0)					
	Other or prefer not to answer	2 (16.7)	0 (0)	10 (83.3)		12 (100)					
**Age (years)**											.008^b^
	35-39	10 (12.2)	15 (18.3)	57 (69.5)		82 (100)					
	40-49	27 (19.1)	20 (14.2)	94 (66.7)		141 (100)					
	50-59	31 (20.8)	15 (10.1)	103 (69.1)		149 (100)					
	60-69	54 (32.7)	22 (13.3)	89 (53.9)		165 (100)					
	70-79	19 (27.1)	10 (14.3)	41 (58.6)		70 (100)					
**Highest level of education attained**											.65
	High school diploma, GED^c^, or less	36 (20.6)	25 (14.3)	114 (65.1)		175 (100)					
	Technical training or certificate	11 (23.9)	8 (17.4)	27 (58.7)		46 (100)					
	Some college or associates degree	43 (21.4)	27 (13.4)	131 (65.2)		201 (100)					
	College degree	29 (26.1)	13 (11.7)	69 (62.2)		111 (100.0)					
	Graduate or professional degree	22 (29.7)	9 (12.2)	43 (58.1)		74 (100)					
**Annual household income (US $)**											.83
	<20,000	25 (22.9)	20 (18.3)	64 (58.7)		109 (100)					
	20,000-35,000	23 (20)	16 (13.9)	76 (66.1)		115 (100)					
	35,000-50,000	29 (28.2)	8 (7.8)	66 (64.1)		103 (100.0)					
	50,000-75,000	24 (22)	18 (16.5)	67 (61.5)		109 (100)					
	75,000-100,000	20 (27.4)	7 (9.6)	46 (63)		73 (100)					
	>100,000	16 (19.5)	10 (12.2)	56 (68.3)		82 (100)					
	Unknown or prefer not to answer	4 (25)	3 (18.8)	9 (56.2)		16 (100)					
**Insurance type**											.39
	Private	50 (21.8)	28 (12.2)	151 (65.9)		229 (100)					
	Government	74 (24.6)	40 (13.3)	187 (62.1)		301 (100)					
	Uninsured	6 (14)	9 (20.9)	28 (65.1)		43 (100)					
	Other insurance or more than 1 policy	11 (32.4)	5 (14.7)	18 (52.9)		34 (100)					
**Relationship statu**s											.29
	Single	34 (26.4)	19 (14.7)	76 (58.9)		129 (100)					
	Married	56 (20.9)	32 (11.9)	180 (67.2)		268 (100)					
	Separated or divorced	36 (24)	20 (13.3)	94 (62.7)		150 (100)					
	Widowed	15 (26.3)	10 (17.5)	32 (56.1)		57 (100)					
	Other or unknown or prefer not to answer	0 (0)	1 (33.3)	2 (66.7)		3 (100)					
**Employment status**											.01^b^
	Full-time	37 (17.9)	32 (15.5)	138 (66.7)		207 (100)					
	Part-time	19 (26.8)	8 (11.3)	44 (62)		71 (100)					
	Not working for pay or unemployed	26 (21.5)	7 (5.8)	88 (72.7)		121 (100)					
	Retired	57 (28.5)	34 (17)	109 (54.5)		200 (100)					
	Other or unknown or prefer not to say	2 (25)	1 (12.5)	5 (62.5)		8 (100)					
**Country of birth**											.55
	United States	128 (23.1)	79 (14.2)	348 (62.7)		555 (100)					
	Outside of the United States	13 (25)	3 (5.8)	36 (69.2)		52 (100)					
**Length of US residency**											.27
	Less than 15 years	3 (15)	2 (10)	15 (75)		20 (100)					
	More than 15 years	138 (23.5)	80 (13.6)	369 (62.9)		587 (100)					

^a^N/A: not applicable.

^b^*P*<.05.

^c^GED: graduate educational diploma.

**Table 5:** Nonparametric analysis of factors influencing clicking on a decision aid seen on social media: social media trustworthiness and demographic insights.

Variable			Likelihood of clicking on a decision aid seen on social media							Respondents, n (%)			*P* value	
			Unlikely		Neutral		Likely							
					
Total			151 (24.88)		101 (16.64)		355 (68.48)		607 (100)			N/A^a^		
**Trustworthiness of social media**													<.001^b^	
	Untrustworthy	103 (37.2)		49 (17.7)		125 (45.1)		277 (100)						
	Neutral	42 (16.3)		46 (17.9)		169 (65.8)		257 (100)						
	Trustworthy	6 (8.2)		6 (8.2)		61 (83.6)		73 (100)						
**Ethnicity**													.72	
	Hispanic	142 (25.5)		89 (16)		325 (58.5)		556 (100)						
	Non-Hispanic	9 (18.8)		11 (22.9)		28 (58.3)		48 (100)						
	Unknown or prefer not to answer	0 (0)		1 (33.3)		2 (66.7)		3 (100)						
**Race**													.19	
	White	121 (25.2)		79 (16.5)		280 (58.3)		480 (100)						
	Black	22 (30.6)		13 (18.1)		37 (51.4)		72 (100)						
	Asian	3 (11.1)		4 (14.8)		20 (74.1)		27 (100)						
	Native American or Alaskan Native	3 (18.8)		4 (25)		9 (56.2)		16 (100)						
	Native Hawaiian or Pacific Islander	0 (0)		0 (0)		0 (0)		0 (0)						
	Other or prefer not to answer	2 (18.2)		1 (9.1)		8 (72.7)		12 (100)						
**Age (years)**													.06	
	35-39	12 (14.6)		16 (19.5)		54 (65.9)		82 (100)						
	40-49	28 (19.9)		25 (17.7)		88 (62.4)		141 (100)						
	50-59	35 (23.5)		26 (17.4)		88 (59.1)		149 (100)						
	60-69	53 (32.1)		25 (15.2)		87 (52.7)		165 (100)						
	70-79	23 (32.9)		9 (12.9)		38 (54.3)		70 (100)						
**Highest level of education attained**													.48	
	High school diploma, GED^c^, or less	38 (21.7)		33 (18.9)		104 (59.4)		175 (100)						
	Technical training or certificate	12 (26.1)		9 (19.6)		25 (54.3)		46 (100)						
	some college or associates degree	47 (23.4)		34 (16.9)		120 (59.7)		201 (100)						
	College degree	28 (25.2)		15 (13.5)		68 (61.3)		111 (100)						
	Graduate or professional degree	26 (35.1)		10 (13.5)		38 (51.4)		74 (100)						
**Annual household income (US $)**														.43
	<20,000	30 (27.5)		22 (20.2)		57 (52.3)		109 (100)						
	20,000-35,000	24 (20.9)		20 (17.4)		71 (61.7)		115 (100)						
	35,000-50,000	28 (27.2)		14 (13.6)		61 (59.2)		103 (100)						
	50,000-75,000	26 (23.9)		24 (22)		59 (54.1)		109 (100)						
	75,000-100,000	23 (31.5)		6 (8.2)		44 (60.3)		73 (100)						
	>100,000	16 (19.5)		11 (13.4)		55 (67.1)		82 (100)						
	Unknown or prefer not to answer	4 (25)		4 (25)		8 (50)		16 (100)						
**Insurance type**														.54
	Private	44 (21.3)		38 (18.4)		125 (60.4)		229 (100)						
	Government	13 (18.3)		13 (18.3)		45 (63.4)		301 (100)						
	Uninsured	28 (23.1)		15 (12.4)		78 (64.5)		43 (100)						
	Other insurance or more than 1 policy	63 (31.5)		34 (17)		103 (51.5)		34 (100)						
**Relationship status**														.37
	Single	39 (30.2)		18 (14)		72 (55.8)		129 (100)						
	Married	58 (21.6)		44 (16.4)		166 (61.9)		268 (100)						
	Separated or divorced	40 (26.7)		25 (16.7)		85 (56.7)		150 (100)						
	Widowed	14 (24.6)		13 (22.8)		30 (52.6)		57 (100)						
	Other or unknown or prefer not to answer	0 (0)		1 (33.3)		2 (66.7)		3 (100)						
**Employment status**														.046^b^
	Full-time	44 (21.3)		38 (18.4)		125 (60.4)		207 (100)						
	Part-time	13 (18.3)		13 (18.3)		45 (63.4)		71 (100)						
	Not working for pay or unemployed	28 (23.1)		15 (12.4)		78 (64.5)		121 (100)						
	Retired	63 (31.5)		34 (17)		103 (51.5)		200 (100)						
	Other or unknown or prefer not to say	3 (37.5)		1 (12.5)		4 (50)		8 (100)						
**Country of birth**													>.99	
	United States	137 (24.7)		94 (16.9)		324 (58.4)		555 (100)						
	Outside of the United States	14 (26.9)		7 (13.5)		31 (59.6)		52 (100)						
**Length of US residency**														.19
	Less than 15 years	2 (10)		4 (20)		14 (70)		20 (100)						
	More than 15 years	149 (25.4)		97 (16.5)		341 (58.1)		587 (100)						

^a^N/A: not applicable.

^b^*P*<.05.

^c^GED: graduate educational diploma.

**Figure 1:**
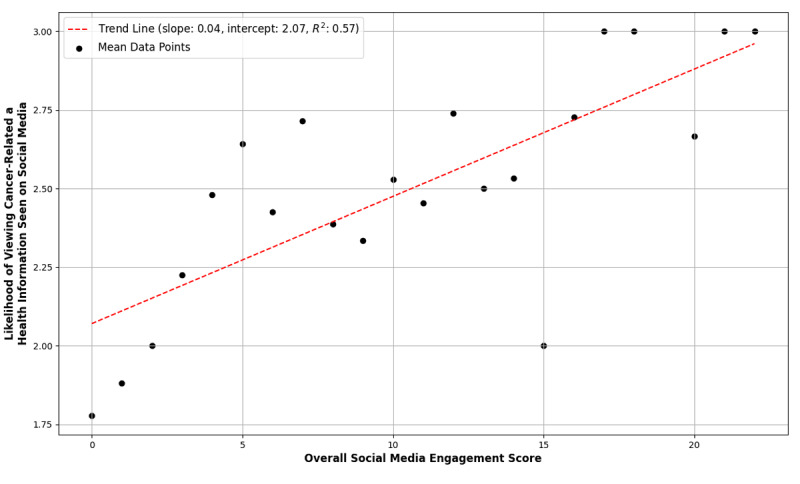
Mean likelihood of viewing cancer-related health information seen on social media by “Overall Social Media Engagement Score.”.

**Figure 2:**
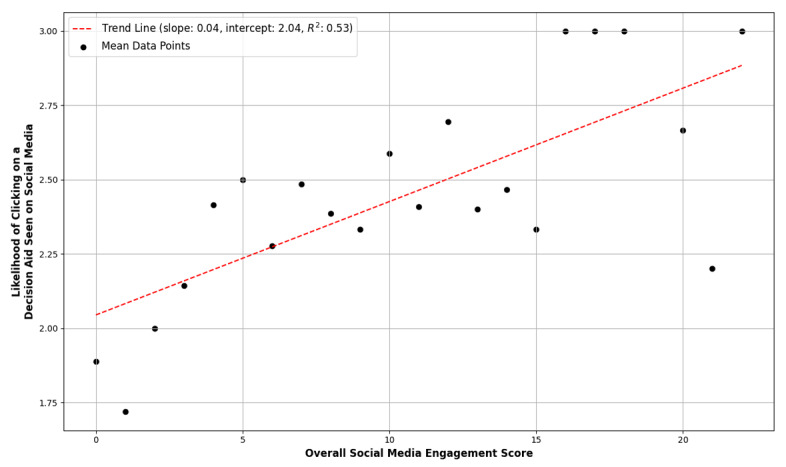
Mean likelihood of clicking on a decision aid seen on social media by “Overall Social Media Engagement Score.”.

The “Overall Social Media Engagement Score” was associated with increased likelihood of viewing cancer treatment-related information (Spearman ρ=0.210, *P<.*001; Figure 1). For instance, among those with an engagement score of 1, only 10 out of 25 (40%) were likely to view cancer information, whereas among those with an engagement score of 8, 28 out of 44 (63.6%) were likely. In addition, the engagement score was also associated with accessing a decision aid on social media (Spearman ρ=0.203, *P*<.001; Figure 2). Among respondents with an engagement score of 1, only 8 out of 25 (32%) were likely to click on the decision aid, whereas for those with a score of 8, 25 out of 44 (56.8%) indicated they were likely.

Age, employment, and perceptions of trustworthiness of social media were included as covariates in the ordinal regression models after significant multicollinearity was ruled out variance inflation factor (VIF≤1.2). The model fit for estimating the likelihood of clicking on decision aids was significant (*χ*²_9_=60.7, *P*<.001, Nagelkerke *R*²=0.113). Respondent perception of the trustworthiness of social media for health information was a significant predictor. Compared with those who found social media trustworthy, respondents who considered social media “untrustworthy” (β=–1.826, Wald *χ*²=29.14, *P*<.001) or “neutral” (β=–0.926, Wald *χ*²=7.22, *P*<.007) were less likely to click. Age category (*P*=.59) and employment status (*P*=.29) were not significant predictors.

The model for viewing cancer-related health information was also significant (χ²_9_==70.4, *P*<.001, Nagelkerke *R*²=0.133). Relative to those rating social media as trustworthy, those rating it as “untrustworthy” had significantly reduced odds of viewing cancer-related health information (β=–1.680, Wald *χ*²=24.31, *P*<.001) while the reduction in likelihood of those rating it “neutral” did not reach statistical significance (β=–0.581, Wald *χ*²=2.74, *P*=.10). Once again, age (*P*=.35) and employment status (*P*=.22) were not significant predictors.

## Discussion

### Principal Findings

The expanding role of social media in health information dissemination underscores a shift in public health communication. The internet is the most frequently reported source of information for individuals with cancer, especially among women, aligning with our findings [32]. To our knowledge, this is the first study to examine factors influencing engagement with decision aids and cancer-related information amongst women using social media platforms.

These study findings suggest that social media holds potential as a platform for the effective dissemination of cancer decision aids to women. Overall social media usage was high, with almost half of the respondents reporting moderate-high to high engagement. We found that about two-thirds of participants searched for cancer-related information, and nearly half of those used the internet to seek such information for themselves in the past year. Usage patterns varied across platforms: while Facebook emerged as the most used platform, WhatsApp was perceived as the most trustworthy source for health information among our respondents.

Most respondents expressed interest in engaging with cancer treatment information or clicking on a decision aid via social media. Higher frequency of social media use correlated with a higher likelihood of interacting with cancer-related content and decision aids online. In addition, trust in social media appears to be a mediating factor in the relationship between demographics and engagement with cancer information on social media. While younger participants and those who worked full time were more likely to view cancer-related information and click on a decision aid, this effect may be a function of their higher likelihood of trusting social media.

### Comparison to Previous Work

Integrating two primary concepts of this study, trust in social media and the likelihood of engaging with health-related content, our findings suggest that individuals who perceive social media as a trustworthy source of health information are more likely to interact with cancer-related treatment information, regardless of their demographic. Numerous consumer studies have highlighted the importance of source credibility in engagement [33-35]. We recognize that trust is a multifaceted construct that is objectively hard to evaluate because it is influenced by factors including demographics, past experiences, and societal and cultural norms [36]. Future studies should focus efforts on better understanding their impact on health information engagement.

Recently, a study by Fridman et al [37] analyzed social media usage and trust in health information among patients with cancer and caregivers, focusing on demographic factors linked to social media use for medical decisions. They also found that a substantial proportion of patients with cancer and caregivers trust social media for health information. Factors associated with higher trust and engagement with social media tools included young age, Black race, and lower education levels. This is consistent with our findings that support trust as a motivating factor for engagement with social media in a medical decision-making context.

Our study was designed to identify trends and general usage patterns across several social media platforms. Facebook (Meta) was the most popular platform among our survey respondents, with 84.2% reporting usage, aligning with 2023 national data indicating that 69% of consumers use Facebook, making it the most used social media site [38]. This is consistent with findings from a recent study which found that Facebook was the most frequently used social media platform for health behavior interventions [39]. However, Facebook is not perceived as being as trustworthy as some of the other platforms. Given the relationship between perceptions of trustworthiness and the likelihood of a respondent using health decision aids on social media, popularity should not be the only factor guiding dissemination. The demographic profile of users also continues to evolve. For example, although less widely used overall, WhatsApp (Meta) is increasingly popular among Latino or Hispanic populations in the United States [40]. These trends highlight the importance of understanding variations in platform use amongst different cohorts when considering platform selection.

In addition, consideration of platforms that can deliver information in diverse formats (eg, text, video, photos, and polls) is important, as each platform’s design and interaction style may be better suited for specific sub-audiences. Future research should focus on exploring platform-specific strategies for health information delivery, especially as new platforms emerge (eg, Bluesky [Bluesky PBLLC] and Threads [Meta]) and others become less frequent.

Moreover, the frequency of social media use has a significant impact on the likelihood of engaging with decision aids or accessing cancer-related health information. Frequent social media users may be more likely to perceive others on these platforms as having integrity and competence. They may also report stronger connections with and greater concern for other network users [41]. Frequency of social media use can significantly influence user interactions, such as clicking behavior [42]. However, our Spearman correlation analysis, which focused solely on frequency, accounted for only about 4% of the variance in viewing and clicking behaviors. This suggests that trust, along with other unmeasured factors, likely plays a critical role in these engagement dynamics.

### Strengths and Limitations

This exploratory study provides insight into the use of decision aids for health information on social media and highlights the key role of trust. It is an important stepping stone for future research assessing online health behavior among female patients in cancer. A key strength of our study is the large sample size (N=607) and the inclusion of a cohort that is fairly representative of United States female population demographics based on census data, enhancing the generalizability of our findings. In addition, by recruiting a female-only cohort, our study offers a more nuanced understanding of preferences and engagement patterns among women, which can inform the development of female-specific cancer decision aids tailored for social media.

As a cross-sectional survey-based study, this study was not designed to explore the many complex, nuanced factors associated with online use behavior. First, the survey, although informed by widely used and nationally developed surveys, was not pretested or pilot-tested for face validity. This lack of initial testing may have been associated with increased confusion among participants and could have influenced responses and overall participation in the survey. Relatedly, we were close but unable to meet the overall target population size of over 660 after the application of exclusion criteria. Second, as an internet-based survey, it was subject to selection bias. Participation required English proficiency, internet access, and the ability to navigate an online survey. While incorporating multilanguage options can be considered, facility and comfort with the internet would still be required. Third, we were unable to recruit a racially diverse population that would be matched with corresponding US census data. For instance, the proportion of Hispanic respondents was nearly half of the intended goal (7.9% vs 16%). However, there was more variation in age, education, and income distribution of respondents. Future efforts in larger populations should focus on targeting underrepresented demographics via other survey distribution platforms and recruitment strategies.

Fourth, regarding statistical analysis, although a multivariable regression was performed, our regression models only accounted for roughly 10%-15% of the likelihood of viewing health information or clicking on a decision aid seen on social media. Moreover, potential influences from unmeasured factors, such as medical conditions or personality traits, further complicated attempts to understand these complex dynamics [43,44].

### Future Directions

This study offers an initial insight into factors influencing online health information and highlights the role of trust. Future research should explore the potential of social media for the delivery of online decision aids specifically designed for patients seeking cancer information. Our study points to the need for pilot testing health decision tools within the target demographic to help with tool optimization and reliability of findings. Trust is a nuanced concept, and efforts should focus on ways to better quantify and distinguish between trust in both social media platforms and online materials.

Once decision aids have been refined for their target population, continued efforts should consider strategies to promote adoption and optimize engagement. Partnering with reputable health organizations, featuring endorsements from trusted medical professionals, and using verified accounts for content delivery can all be considered. Examining platform-specific formats, such as interactive content on Facebook or visual aids on Instagram, could help increase diffusion. Presenting clear, evidence-based information in user-friendly, visually engaging formats (eg, infographics or explainer videos) may further increase credibility. Incorporating interactive features that allow users to connect with health care providers or support groups on social media could also render greater trust and engagement. Research on these trust-building strategies would offer valuable insight into optimizing social media as a reliable and accessible channel for health information dissemination.

## Data Availability

The datasets generated or analyzed during this study are available from the corresponding author on a reasonable request.

## Appendix

Multimedia Appendix 1 Survey Used in Study.

Multimedia Appendix 2 Checklist for Reporting Results of Internet E-Surveys (CHERRIES).

## Abbreviations

CHERRIES: Checklist for Reporting Results of Internet E-Surveys
SDM: shared decision-making
VIF: variance inflation factor
