# Correction: Social Media as a Platform for Cancer Care Decision-Making Among Women: Internet Survey-Based Study on Trust, Engagement, and Preferences

**DOI:** 10.2196/87683

**Published:** 2026-02-13

**Authors:** Anna Rose Johnson, Grace Anne Longfellow, Clara N Lee, Benjamin Ormseth, Gary B Skolnick, Mary C Politi, Yonaira M Rivera, Terence Myckatyn

**Affiliations:** 1 Division of Plastic and Reconstructive Surgery Department of Surgery Washington University School of Medicine Saint Louis, MO United States; 2 Department of Surgery, Lineberger Comprehensive Cancer Center University of North Carolina at Chapel Hill Chapel Hill, NC United States; 3 Department of Plastic Surgery University of Texas Southwestern Medical Center Dallas, TX United States; 4 Division of Public Health Sciences, Department of Surgery Washington University School of Medicine Saint Louis, MO United States; 5 Department of Communication School of Communication & Information Rutgers University New Brunswick, NJ United States

In “Social Media as a Platform for Cancer Care Decision-Making Among Women: Internet Survey-Based Study on Trust, Engagement, and Preferences. JMIR Cancer” [1], the authors noted one omission.

A "Funding" section has been added as follows:

Research reported in this publication was supported by the National Cancer Institute of the National Institutes of Health under Award Number R01CA276408. The content is solely the responsibility of the authors and does not necessarily represent the official views of the National Institutes of Health.

The correction will appear in the online version of the paper on the JMIR Publications website, together with the publication of this correction notice. Because this was made after submission to PubMed, PubMed Central, and other full-text repositories, the corrected article has also been resubmitted to those repositories.
