# Supplementary material for: Social Media as a Platform for Cancer Care Decision-Making Among Women: Internet Survey-Based Study on Trust, Engagement, and Preferences
Source: JMIR Cancer. 2025 Mar 5;11:e64724. doi: 10.2196/64724 (PMC11923483; doi:10.2196/64724)
Supplement: Multimedia Appendix 1 [file cancer_v11i1e64724_app1.docx]

**Appendix A.**

Please answer the following survey questions to the best of your ability.

Age Screening Question:

What is your age?

- 18-34
- 35-55
- 56-75
- 75+

**Health-related information behaviors**

1. Have you ever looked for information about health or medical topics from any source?

- Yes
- No

1. The most recent time you looked for information about health or medical topics, where did you go **first**?

- Books
- Brochures, pamphlets, etc.
- Cancer organization
- Family
- Friend/Co-worker
- Doctor or healthcare provider
- Internet
- Library
- Magazines
- Newspapers
- Telephone information number
- Complementary, alternative, or unconventional practitioner

1. The most recent time you looked for information about health or medical topics, who was it for?

- Myself
- Someone else
- Both myself and someone else

1. Have you ever looked for information about **cancer** from any source?

- Yes
- No

1. In the past 12 months, have you used the **Internet** to look for cancer information for yourself?

- Yes
- No

*Attention check question*

Please select 'Strongly agree' to show you are paying attention to this question.

- Strongly disagree
- Disagree
- Agree
- Strongly agree

**Sources of health information**

1. Which of the following sources have you **used** in the last month as a source of news or information about health topics? Check all that apply:

- Blogs/personal websites
- Centers for Disease Control and Prevention
- Community/faith leaders
- Online news
- Email
- Family and friends
- Government (Federal, provincial, local)
- Health professionals (Scientists, doctors, public health officials)
- Podcasts
- Print media (Newspapers, magazines, etc.)
- Private messaging apps (WhatsApp, Messenger, WeChat, etc.)
- Radio
- Social media (Facebook, Weibo, Twitter, Instagram, Weibo, Pinterest, etc.)
- TV (Commercials, news programs, entertainment programs, etc.)
- Video sharing sites (YouTube, TikTok)
- World Health Organization

1. What sources do you **trust** to get accurate health information? Check all that apply:

- Blogs/personal websites
- Centers for Disease Control and Prevention
- Community/faith leaders
- Online news
- Email
- Family and friends
- Government (Federal, provincial, local)
- Health professionals (Scientists, doctors, public health officials)
- Podcasts
- Print media (Newspapers, magazines, etc.)
- Private messaging apps (WhatsApp, Messenger, WeChat, etc.)
- Radio
- Social media (Facebook, Weibo, Twitter, Instagram, Weibo, Pinterest, etc.)
- TV (Commercials, news programs, entertainment programs, etc.)
- Video sharing sites (YouTube, TikTok)
- World Health Organization

1. How trustworthy do you consider the information shared by these sources?

|  | Very untrustworthy | Untrustworthy | Neither untrustworthy or trustworthy | Trustworthy | Very trustworthy |
| --- | --- | --- | --- | --- | --- |
| 1. Scientists, doctors | 1 | 2 | 3 | 4 | 5 |
| 1. Public health officials | 1 | 2 | 3 | 4 | 5 |
| 1. Governments | 1 | 2 | 3 | 4 | 5 |
| 1. Traditional news | 1 | 2 | 3 | 4 | 5 |
| 1. Social media | 1 | 2 | 3 | 4 | 5 |
| 1. Friends and family | 1 | 2 | 3 | 4 | 5 |
| 1. Video sharing sites | 1 | 2 | 3 | 4 | 5 |
| 1. Community/Faith leaders | 1 | 2 | 3 | 4 | 5 |
| 1. Online forum/discussion board | 1 | 2 | 3 | 4 | 5 |
| 1. Online news | 1 | 2 | 3 | 4 | 5 |

**Social media use**

1. Where do you access your social media accounts? (Select all that apply)

- Computer/laptop
- iPad or tablet
- Smartphone (iPhone, Android, etc.)

1. What social media sites do you use? Check all that apply:
   1. Facebook
   2. Twitter
   3. Instagram
   4. YouTube
   5. WhatsApp
   6. Other ___________
   7. I do not use social media
2. How often do you check Facebook?

- Multiple times a day
- Once a day
- At least 3 times a week
- Less than 3 times a week

1. Why do you use Facebook? (Select all that apply)

- Social interactions
- To search for information
- To pass time/Boredom
- Entertainment
- Relaxation
- Communication tool
- To express opinions
- Convenience
- To share information
- To see what others are doing
- Advocacy
- To share/maintain my cultural identity
- Other: __________________________________________________________

1. How often do you check Twitter?

- Multiple times a day
- Once a day
- At least 3 times a week
- Less than 3 times a week

1. Why do you use Twitter? (Select all that apply)

- Social interactions
- To search for information
- To pass time/Boredom
- Entertainment
- Relaxation
- Communication tool
- To express opinions
- Convenience
- To share information
- To see what others are doing
- Advocacy
- To share/maintain my cultural identity
- Other:

1. How often do you check Instagram?

- Multiple times a day
- Once a day
- At least 3 times a week
- Less than 3 times a week

1. Why do you use Instagram? (Select all that apply)

- Social interactions
- To search for information
- To pass time/Boredom
- Entertainment
- Relaxation
- Communication tool
- To express opinions
- Convenience
- To share information
- To see what others are doing
- Advocacy
- To share/maintain my cultural identity
- Other:

1. How often do you check YouTube?

- Multiple times a day
- Once a day
- At least 3 times a week
- Less than 3 times a week

1. Why do you use YouTube? (Select all that apply)

- Social interactions
- To search for information
- To pass time/Boredom
- Entertainment
- Relaxation
- Communication tool
- To express opinions
- Convenience
- To share information
- To see what others are doing
- Advocacy
- To share/maintain my cultural identity
- Other:

1. How often do you check WhatsApp?

- Multiple times a day
- Once a day
- At least 3 times a week
- Less than 3 times a week

1. Why do you use WhatsApp? (Select all that apply)

- Social interactions
- To search for information
- To pass time/Boredom
- Entertainment
- Relaxation
- Communication tool
- To express opinions
- Convenience
- To share information
- To see what others are doing
- Advocacy
- To share/maintain my cultural identity
- Other:

1. How often do you check TikTok?

- Multiple times a day
- Once a day
- At least 3 times a week
- Less than 3 times a week

1. Why do you use TikTok? (Select all that apply)

- Social interactions
- To search for information
- To pass time/Boredom
- Entertainment
- Relaxation
- Communication tool
- To express opinions
- Convenience
- To share information
- To see what others are doing
- Advocacy
- To share/maintain my cultural identity
- Other:

1. How often do you check the “other” social media site that you reported?

- Multiple times a day
- Once a day
- At least 3 times a week
- Less than 3 times a week

1. Why do you use the “other” social media site that you reported? (Select all that apply)

- Social interactions
- To search for information
- To pass time/Boredom
- Entertainment
- Relaxation
- Communication tool
- To express opinions
- Convenience
- To share information
- To see what others are doing
- Advocacy
- To share/maintain my cultural identity
- Other:

1. Imagine that you or a loved one were making a decision about cancer treatment. If you saw information about cancer treatment posted on social media, how likely would you be to view it**?**

|  |  |  |  |  |  |  |
| --- | --- | --- | --- | --- | --- | --- |
| 1  Extremely unlikely | 2 | 3 | 4 | 5 | 6 | 7  Extremely likely |

1. Imagine that you or a loved one were making a decision about cancer treatment. If you saw a decision aid (tool that helps people make decisions) about cancer treatment posted on social media, how likely would you be to click on that decision tool**?**

|  |  |  |  |  |  |  |
| --- | --- | --- | --- | --- | --- | --- |
| 1  Extremely unlikely | 2 | 3 | 4 | 5 | 6 | 7  Extremely likely |

*Attention check question*

The following question is to verify that you are a real person. Which of the following is a vegetable?

- Egg
- Salmon
- Broccoli
- Cheeseburger
- Pizza
- Milk

**Demographic questions**

The next two questions are about your race and ethnicity. To understand more about people who use online health information, we would like to record this using specific categories. First, are you Latino/a/x or Hispanic?

- Yes
- No
- Prefer not to answer

Second, which category or categories best describes you? Mark all answers that apply.

- African American or Black
- Asian
- Caucasian or White
- Native American or Alaskan Native
- Native Hawaiian or other Pacific Islander
- Other (Specify)
- Prefer not to answer

What is your age?

18-29

30-49

50-64

- 65 or older

What is the highest level of formal education you have completed?

- Less than high school
- Some high school
- A high school diploma or GED
- Technical training or certificate
- Some years of college or Associates Degree
- A college degree (example: BA, BS, BFA)
- A graduate or professional degree (example: master’s degree, PhD, MD, JD)
- Prefer not to answer

What is your annual income, meaning the total pre-tax income from all sources earned in the past year?

- Less than $20,000
- At least $20,000 but less than $35,000
- At least $35,000 but less than $50,000
- At least $50,000 but less than $75,000
- At least $75,000 but less than $100,000
- $100,000 or more
- Prefer not to answer

Are you currently covered by any of the following types of health insurance or health coverage plans? Please select all that apply.

- Private insurance purchased through a current or former employer/union or directly from an insurance company or marketplace.
- Government insurance like Medicare or Medicaid
- No insurance of any type
- Any other type of health insurance coverage or health coverage plan (please specify):

What is your marital status?

- Single
- Married
- Separated
- Divorced
- Widowed
- Prefer not to answer

What is your employment status? You can check all that apply

- I work full time
- I work part time
- I am not working for pay/unemployed
- I am a student
- I am retired
- Prefer not to say

Where were you born?

- United States (including Puerto Rico)
- Outside of the United States.

How long have you lived in the United States?

- 1-5 years
- 6-10 years
- 11-15 years
- 15+ years
